# Tuning dopant incorporation in tin oxide thin films using a methanol–water solvent for photovoltaic and water splitting applications

**DOI:** 10.1039/d5ra08325g

**Published:** 2026-01-30

**Authors:** Musarrat Zahra, Shafqat Hussain, Khurram Shehzad, Ahsan Jamal, Kashif Yaqub, Muhammad Rehan, Mohsin Ali Raza Anjum, Jaweria Ambreen, Muhammad Saifullah

**Affiliations:** a Department of Chemistry, COMSATS University Islamabad Park Road Islamabad 45550 Pakistan Jaweria.ambreen@comsats.edu.pk; b Physics Division (PD), Pakistan Institute of Nuclear Science and Technology (PINSTECH) Islamabad Pakistan; c Central Analytical Facility Division (CAFD), Pakistan Institute of Nuclear Science and Technology (PINSTECH) Islamabad Pakistan; d Chemistry Division (CD), Pakistan Institute of Nuclear Science and Technology (PINSTECH) P. O. Box 45650, Nilore Islamabad Pakistan Saifi.551@gmail.com; e LINAC Project, Pakistan Institute of Nuclear Science and Technology (PINSTECH) Nilore Islamabad 45650 Pakistan; f Photovoltaic Research Department, Korea Institute of Energy Research (KIER) Daejeon South Korea

## Abstract

Fluorine-doped tin oxide (FTO) thin films serve as a good substrate for photovoltaic and electrochromic devices due to their outstanding optoelectronic features. The current study probes the profound impact of systematically varying the F/Sn molar ratio over a wide range on optoelectronic properties of spray pyrolyzed FTO thin films, leading to optimization of the F/Sn ratio for the methanol-to-water solvent system (9 : 1 v/v). A comprehensive suite of characterizations reveals that all FTO samples exhibit the tetragonal SnO_2_ phase with a preferred (200) orientation, while increasing the F/Sn ratio up to 2.8 (M2.8) enhances the crystallite size. Distinctive grain morphologies, including pyramidal, polygon-type, and bud-like structures, are observed across all samples. Optical transparency in the visible spectrum increases, reaching an impressive 79% for M2.8. Hall effect measurement confirms a significant rise in carrier concentration, with M2.8 showing the highest value. Additionally, M2.8 achieves the lowest sheet resistance of 4.4 Ω □^−1^ and an excellent figure of merit of 0.02 Ω^−1^ along with high thermochemical stability, making it an optimized candidate for high-performance applications. The practicability of optimized samples is demonstrated through their successful integration into perovskite solar cells, achieving a commendable power conversion efficiency of 15% under standard AM1.5 G illumination. The performance of indigenous substrates in water-splitting applications is comparable to commercial FTO thin films, underscoring their versatility. These findings emphasize the critical role of fine-tuning the F/Sn molar ratio for a specific solvent system to achieve superior optoelectronic properties of FTO thin films and pave the way for their widespread adoption in next-generation energy and electronic applications.

## Abstract 2

Fluorine-doped tin oxide substrates, with optimized F : Sn ratio in methanol–water solvent, shown high figure of merit of 0.02 Ω^−1^ and demonstrated a good potential in photovoltaic and water splitting applications.

## Graphical abstract

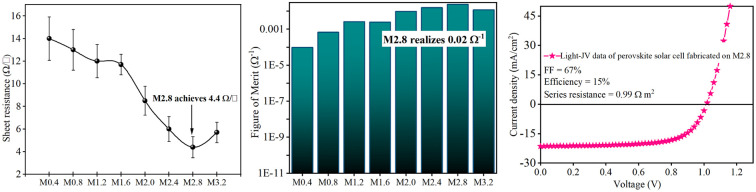

## 1. Introduction

Many optoelectronic devices utilize transparent conducting oxides (TCOs) as the essential structural components owing to their high conductivity and good transparency through the visible range.^1–6^ TCOs are widely studied due to their wide bandgap (*E*_g_) semiconducting properties, ease of deposition on various substrates, and ability to tailor physical characteristics to modify optoelectronic properties.^7^ Various TCOs have been reported to have good optoelectronic properties, including tin-doped indium oxide (ITO),^8,9^ antimony-doped tin oxide,^10,11^ and fluorine-doped tin oxide (FTO). However, FTOs are largely considered efficient substrates owing to their robust capability of withstanding intense thermochemical conditions, excellent optoelectronic properties, and low cost.^4^ FTO comprises tin oxide (SnO_2_) as a host lattice to which fluoride (F^−^) ions are doped. SnO_2_ is an n-type semiconducting material due to non-stoichiometry resulting from intrinsic oxygen vacancy defects, which donate electrons to the conduction band.^12–16^ Intrinsic SnO_2_ possesses a relatively high resistivity, limiting its application as a TCO.^13^ Doping with a suitable impurity can enhance the conductivity of SnO_2_ by providing additional electrons in its crystal structure.^14^ Amongst many investigated cationic (*i.e.*, Sb, Mo, and Nb, *etc.*)^14–16^ and anionic (halide) dopants, F^−^ is favorable owing to its comparable radius (1.40 Å) to the oxide ion (O^2−^ = 1.33 Å), thereby facilitating the easy substitution of O^2−^ with F^−^ in the SnO_2_ lattice.

Doping of the SnO_2_ lattice with low-valent anions like F^−^, which replaces O^2−^, furnishes one extra electron. These electrons from the pool in the crystal structure first occupy the empty 2p states of substituted fluorine, and the remaining electrons fill the conduction band of tin, consequently enhancing conductivity without compromising stability.^17,18^ This increases the number of charge carriers and enhances the material's conductivity.^19,20^ The FTO thin film also adheres well to various substrates, resulting in high chemical and electrical stability, as well as good physical resilience under corrosive and thermal testing conditions.^21,22^ Amongst several deposition techniques, the chemical spray pyrolysis (CSP) is the simplest and most economical method, which involves spraying the precursor solution on the preheated substrate. The precursor solution, composed of solvents and precursor salts, is sprayed on the preheated substrate, ensuring the decomposition of these salts to form an FTO thin layer on the substrate. The interaction of precursor salts with the solvents is crucial in determining spray dynamics and ultimately influences the formation of SnO_2_ nuclei. This provides a way to regulate the physicochemical characteristics and, consequently, optoelectronic traits of the prepared FTO thin films. By optimizing the dopant concentration, several features such as grain size, shape, and surface topography can be tailored to achieve the desired optoelectronic properties of FTO thin films.^23^

Several studies have reported the influence of varying dopant-to-precursor salt concentrations in the spraying solution using different doping agents in diverse solvents and observed their effects on the optoelectronic properties of FTO thin films. Such as Moholkar *et al.*^24^ varied the F/Sn molar ratio from 0.3 to 3.7 using NH_4_F at 350 °C. In an aqueous solution, it was identified that FTO with a 1.25 F/Sn molar ratio had the highest carrier density (*n*) of 24.9 × 10^20^ cm^−3^, and the lowest resistivity (*ρ*) of 3.8 × 10^−4^ Ω cm. Likewise, Thirumoorthi and coworkers^25^ grew FTO thin films by varying the F/Sn molar ratio from 0 to 0.2 at 500 °C employing NH_4_F salt and water as solvents. The optimum quality FTO thin films were achieved at 0.18 F/Sn molar ratio with *n* and *ρ* of 6.9 × 10^20^ cm^−3^ and 2.3 × 10^−4^ Ω cm, respectively. In another study, Ramírez-Amador *et al.*^26^ reported the growth of FTO thin films using NH_4_F at a temperature of 450 °C. In this study, the F/Sn ratio was varied in the range of 0 to 0.85 to find its adequate value in the ethanol and water solvent mixture. The optoelectronic properties, including % transmittance (*T*) and sheet resistance (*R*_s_), were found to be 88.6% and 37.47 Ω □^−1^, respectively, for the best FTO sample with an F/Sn ratio of 0.5. Similarly, Karthick *et al.*^12^ studied the impact of varying F/Sn ratios on the optoelectronic properties of FTO thin films employing HF as a doping agent and water as a solvent. They reported the highest *T* of 75% and lowest *ρ* of 9.0 × 10^−4^ Ω cm for the thin film sample grown with an F/Sn molar ratio of 1.2.

From the above studies, it is clear that the optimal F/Sn molar ratio ranges from 0.18 to 1.25 when water is used as the solvent and NH_4_F as the fluorine doping agent. For HF as a doping agent, the optimal F/Sn molar ratio is approximately seven times lower compared to NH_4_F, indicating that the amount of F^−^ incorporated into SnO_2_ can depend on the type of dopant precursor. However, HF, despite being needed in small quantities to introduce optimal fluorine content in the crystal structure of SnO_2_, is corrosive and can be hazardous while handling. Therefore, NH_4_F is a better choice to dope fluorine into the SnO_2_ lattice. Another important processing parameter is the nature of the solvent, which can also be crucial in determining the necessary F/Sn molar ratio in the spraying solution. For example, solvents with different polarity indices, thermodynamic properties, and physicochemical characteristics can interact differently with NH_4_F. In water-based media, the solvation of NH_4_F is very strong, stabilizing the ions and causing an increased effective concentration of F^−^ species for substitution into the SnO_2_ lattice during pyrolysis. In alcohol-rich media, low solvent polarity weakens NH_4_F solvation, reducing the stability and availability of F^−^ ions for lattice incorporation. Additionally, low-boiling alcohols exacerbate solvent evaporation, resulting in premature salt precipitation and partial F^−^ loss. Meanwhile, their reduced viscosity and surface tension produce relatively smaller droplets with shorter residence times, thereby limiting dopant diffusion during pyrolysis. Collectively, these effects decrease fluorine incorporation efficiency in alcoholic sprays, necessitating higher F/Sn ratios to achieve properties comparable to aqueous systems. In our previous study,^27^ spray-pyrolyzed FTO thin films were prepared using a mixture of methanol and water, with a fixed F/Sn ratio, to examine the effect of solvent on optoelectronic properties. The optimal methanol-to-water ratio was found to be 9 : 1 (v/v). However, the F/Sn ratio in the spraying solution remains a critical parameter in the CSP method and significantly affects the optoelectronic properties of FTO thin films; therefore, it requires detailed investigation for methanol-to-water solvent systems, which has not previously been done to the best of our knowledge.

To fabricate FTO thin films with improved optoelectronic characteristics, this study systematically investigates the impact of varying the F/Sn molar ratio in a much wider range (0.4–3.2) compared with the previous studies in a distinct 9 : 1 (v/v) methanol-to-water solvent system. The findings of this study will help determine the adequate concentration of dopant in the above solvent system to grow FTO thin films with enhanced optoelectronic properties and will be beneficial in understanding the sufficient F/Sn molar ratio for other alcoholic solvents. It is found that increasing the F/Sn ratio up to 2.8 significantly improves the physical properties, including crystallite size and surface topography, as well as optoelectronic characteristics. These results support our proposition that a higher F/Sn ratio is required to optimally dope SnO_2_ in low-boiling alcohol solvents. Finally, the best-optimized FTO substrates are employed in perovskite thin film solar cells and water-splitting applications to demonstrate their good quality and potential for real world applications.

## 2. Materials and methods

FTO thin films are grown on 1.75 mm thick soda-lime glass (SLG) substrates with dimensions of 3 × 3 cm^2^, purchased from the local market. Before proceeding to deposition by chemical spray pyrolysis (CSP) method, SLG is stepwise cleaned with detergent solution, distilled water, acetone, and isopropanol for 15 min each, and finally dried in open air.

The chemicals used for fabricating perovskite solar cell on the optimized FTO substrate are dimethyl formamide (DMF) (M.W. = 73.09 g mol^−1^), dimethyl sulfoxide (DMSO) (M.W. = 78.13 g mol^−1^), PbI_2_ and PbBr_2_ (M.W. = 461.01 g mol^−1^ and 367.01 g mol^−1^, respectively), (2-(3,6-dimethoxy-9*H*-carbazol-9-yl)ethyl)phosphonic acid (MeO-2PACz)^28^ (M.W. = 335.29 g mol^−1^), cesium iodide (M.W. = 259.81 g mol^−1^), 2,9-dimethyl-4,7-diphenyl-1,10-phenanthroline (bathocuproine) (M.W. = 360.45 g mol^−1^),^29^ and buckminsterfullerene (C60) (M.W. = 720.66 g mol^−1^). The perovskite solar cell fabrication procedure is described in our earlier study.^27^

### 2.1. FTO thin film preparation

The simple compressed CSP technique is adopted to fabricate FTO thin films. To prepare the precursor solution for each FTO substrate, SnCl_4_·5H_2_O (M.W. = 350.60 g mol^−1^, Daejung, 98%) and NH_4_F (M.W. = 37 g mol^−1^, BDH, 98%) are dissolved in deionized (DI) water (*i.e.*, 10% by vol. to the total spraying volume), followed by the addition of 175 µL of HCl (M.W. = 36.46 g mol^−1^, Merck, 37%) to ensure the stability of the solution. Afterward, methanol (CH_3_OH, M.W. = 32.04 g mol^−1^, Merck, 98.8%) is mixed (90% by vol.) in the previously prepared solution, followed by minimal stirring to obtain the final spraying solution. The afore-described dissolution method of precursor salts is followed to address the limited solubility of precursor salts in CH_3_OH. Eight different samples are fabricated by varying the concentration of NH_4_F to maintain the F/Sn molar ratios, as listed in Table 1. For preparing an FTO thin film, a 25 mL precursor solution is sprayed on the SLG substrates preheated at 450 °C. The molarity of SnCl_4_·5H_2_O salt in the spraying solution is fixed at 0.2 M, whereas the concentration of NH_4_F is varied from 0.08 to 0.64 M. During deposition, the SLG substrate's temperature is maintained at 450 °C on a hotplate to facilitate pyrolysis. A chromatographic atomizer is employed to spray the precursor solution as a fine mist onto the preheated SLG substrate, positioned 12 cm from the nozzle at an angle of ∼60°. The deposition system is automated using a microcontroller and a relay to minimize human error and enhance reproducibility. The detailed deposition process, along with the schematics, is presented in our previous study.^27^

**Table 1:** Summary of samples prepared in this study with different F/Sn molar ratios in the spraying solution and corresponding labels

Label	M0.4	M0.8	M1.2	M1.6	M2.0	M2.4	M2.8	M3.2
F/Sn molar ratio	0.4	0.8	1.2	1.6	2.0	2.4	2.8	3.2

### 2.2. Electrode preparation for the oxygen evolution reaction

To prepare a graphitic carbon nitride (g-C_3_N_4_)-coated FTO substrate, first, catalyst ink is prepared by sonicating 10 mg of catalyst (synthesized for our previous study)^30^ in a mixture of 1 mL of DI water and ethanol each (1 : 1), and 20 µL of Nafion solution (5%) in a beaker. The working electrode is prepared by manually coating the catalyst suspension onto washed and dried FTO substrates with an area of 1.1 cm^2^ for both indigenous and commercial FTO thin films. After each coating cycle, the electrode is dried at 80 °C, and the process is repeated until the total volume of ink has been deposited. A similar method for developing g-C_3_N_4_-coated carbon felt electrodes was followed in our previous study.^30^

For WO_3_ coating both on indigenously developed and commercial FTO thin films, the coating solution is prepared by dissolving 1.5 g of acetylated peroxotungstic acid (APTA) precursor in 5 mL of ethanol. Subsequently, coating is performed using a dip coater at a speed of 2 mm s^−1^ and a dipping time of 10 s. Five cycles of coating are performed to prepare each substrate. The prepared thin films were then dried in an oven for 1 h at 100 °C, followed by annealing at 250 °C for 90 min to convert APTA into amorphous WO_3_ thin films. The XRD pattern and Raman profile of the prepared electrode are shown in Fig. S1 and S2, respectively.

### 2.3. Characterizations

For structural investigations of the prepared FTO thin films, an X-ray diffractometer EQUINOX 3000 X-ray (XRD) with Cu-Kα radiation (wavelength, *λ* = 1.5406 Å) is employed. To investigate different vibrational modes of SnO_2_ present in the prepared FTO thin films, Raman spectra are acquired in the range 1200–200 cm^−1^ using XploRA™ Plus Raman microscope by Horiba Scientific. Surface morphology and topography of the grown FTO thin films are observed by the scanning electron microscope TESCAN MAIA3 Triglav™, and the atomic force microscopy instrument NANOSURF AG-4410LIESTAL. A Hitachi U-2900 UV-visible spectrometer is used to assess the optical transparency of the prepared thin films in the visible range (400–800 nm, step size = 1 nm). The electrical properties of the deposited FTO thin films are analyzed by colinear four-probe analysis using the current (Keithley 6220) and voltage (Keithley 2182A) sources. For further analyzing the mobility and number of charge carriers in the grown FTO samples, Hall effect measurement is performed in van der Pauw configuration employing a 4200A-SCS parameter analyzer. The potential of the best FTO coated with g-C_3_N_4_ and WO_3_ catalysts for the oxygen evolution reaction is examined using a CorrTest-CS2350M electrochemical workstation in alkaline media (1.0 M KOH) employing a three-electrode setup having Ag/AgCl as the reference electrode, graphite rod as the counter electrode, and the prepared coated FTO substrate as a working electrode. The performance of the perovskite solar cell fabricated on the best FTO substrate developed in this study is assessed under standard AM1.5 G irradiation.1
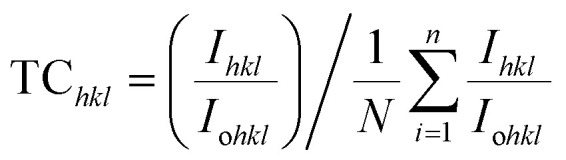
2
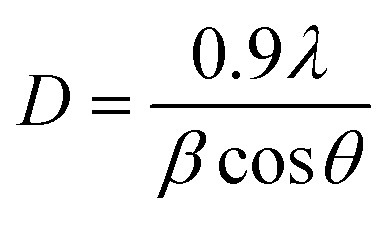
3
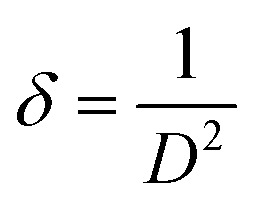
4
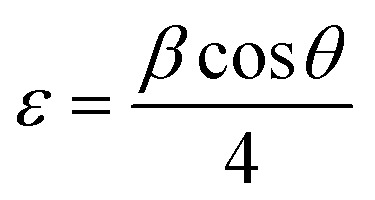

5 (*αhν*) = *A*(*hν* − *E*_g_)^*n*^

6
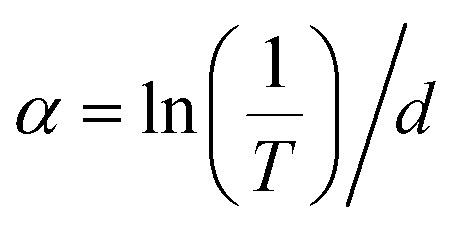

7 *α* = *α*_o_ exp *hν*/*E*_oo_

8 ln *α* = ln *α*_o_ + *hν*/*E*_oo_

9
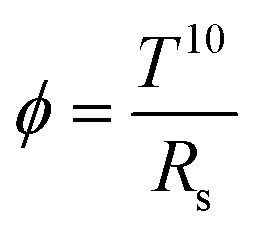

10 *E*_RHE_ = *E*_Ag/AgCl_ + *E*^o^_Ag/AgCl_ + 0.0591 × pH

The texture coefficient of the prepared FTO thin films is determined by XRD analysis using eqn (1). In eqn (1), TC_*hkl*_ represents the texture coefficient of a particular (*hkl*) plane, where, *I*_*hkl*_ is the observed intensity of the plane (*hkl*) in thin films, *I*_o*hkl*_ shows the standard intensity of the corresponding *hkl* plane given in the PDF file, and *N* is the total number of reflections considered for TC analysis. Crystallite size (*D*) is calculated using eqn (2), the well-known Scherrer's formula, where 0.9 is the shape factor, *λ* is the wavelength of X-ray, *i.e.*, 1.5408 Å, *β* is the full-width half-maximum taken in radians, and *θ* is the Bragg's diffraction angle.^25^ The dislocation density (*δ*) and strain (*ε*) in the FTO thin films are estimated by eqn (3) and (4), respectively. The Tauc expression,^31^eqn (5) is used to calculate the *E*_g_ of the deposited FTO thin films, where ‘*α*’ represents the absorption coefficient as a function of wavelength *α*(*λ*), *h* indicates Planck's constant, *E*_g_ is the band gap energy, and *n* is a constant with value 1/2 for direct band gap transitions. The “*α*” in eqn (5) is determined by eqn (6). Urbach tailing is calculated following eqn (7) is defined as the Urbach law, where *α*_o_ is a constant, and *E*_oo_ is the Urbach energy related to the width of the exponential absorption edge. The plot of ln(*α*) as a function of *hν* (eqn (8)) calculates the value of *E*_oo_. The figure of merit (*ϕ*) of the prepared samples is determined employing Haacke's equation, eqn (9), where *T*^10^ is the 10th power of transmittance, and *R*_s_ is the sheet resistance.^32^ In eqn (10), *E*_RHE_ is the potential *versus* RHE, *E*_Ag/AgCl_ is the measured potential *versus* the Ag/AgCl electrode, *E*^o^_Ag/AgCl_ is the standard potential of the Ag/AgCl electrode.^33^

## 3. Results and discussions

### 3.1. Structural, morphological, and topographical investigations

**Fig. 1:**
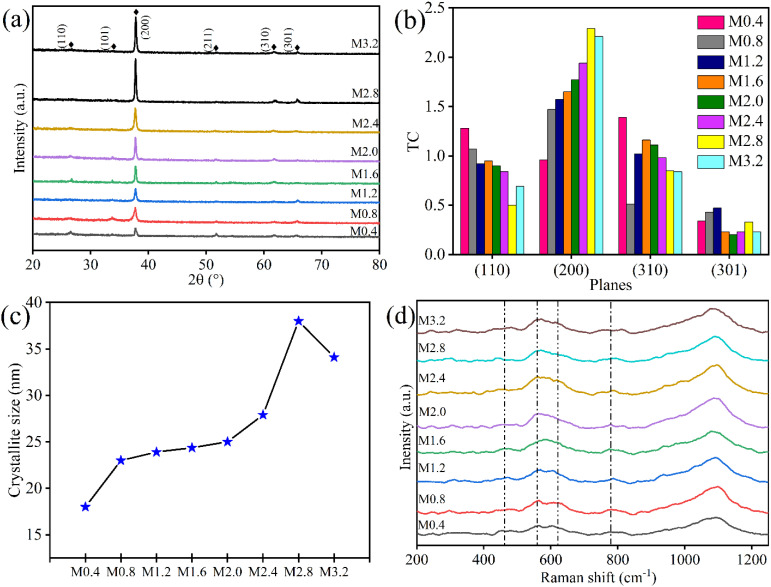
(a) X-ray diffractograms (patterns matching with PDF card no. 00-046-1048, tetragonal phase of SnO_2_) in 2*θ*–*θ* mode, (b) TC values along (110), (200), (310), and (301) planes, (c) crystallite size determined from FWHM of (200) plane, and (d) Raman spectra in the 200–1250 cm^−1^ range of the FTO thin films prepared by varying F/Sn molar ratios from 0.4 to 3.2.

The XRD analysis is employed to investigate the impact of varying F/Sn molar ratios in a methanol : water (9 : 1, v/v) solvent system^27^ on the structural properties of the prepared FTO thin films. The acquired diffractograms of prepared samples in the 2*θ* range of 20°–80° are presented in Fig. 1(a). The diffraction peaks at the 2*θ* positions of 26.5, 33.7, 37.7, 51.7, 61.7, and 65.7°, arising from (110), (101), (200), (211), (310), and (301) planes, respectively, follow the standard PDF card no. 00-046-1048 (tetragonal phase of SnO_2_). The polycrystalline nature of all the prepared FTO thin films is evident from XRD patterns, regardless of the F/Sn molar ratio used for the thin films' growth. Fig. 1(b) depicts the variation in texture coefficients (TC), calculated using eqn (1), for four predominant diffraction peaks observed in XRD patterns. The corresponding standard diffraction intensities described in the PDF card are used for TC determination. Fig. 1(b) reveals that all the FTO samples are preferably oriented along the (200) plane, while M2.8 has the highest deviation from unity, pointing towards its preferential growth along this plane. This suggests that growth along this direction is energetically more favorable.^34^ The gradual increase in the intensity of the (200) plane is linked with an increase in the F/Sn molar ratio. Beyond the F/Sn molar ratio of 2.8, there is no further increase in preferred orientation along the (200) plane, which might be linked to the saturation of F^−^ ions along the plane and, therefore, results in the increased strain that will be discussed later. These findings are in line with the observations made by Moholkar *et al.*,^24^ where it is reported that the increasing F^−^ concentration results in increasingly preferred growth in [200] direction, while a reversal of the trend occurs on further rising F^−^ concentration beyond 20 wt%. The dependence of preferential growth on doping concentration is also reported in the study by Ramaiah *et al.*^34^ The preferred orientation along (200) helps attain high mobility of charge carriers, leading to lowering of the resistance of the FTO thin film.^35,36^

The crystallite size (*D*) in the prepared FTO thin films is determined from the FWHM of the diffraction peak along (200) employing the Debye–Scherrer formula described in eqn (2). Fig. 1(c) illustrates the change in the value of *D* with respect to the F/Sn molar ratio. An increasing trend in *D* is observed with the rise of NH_4_F concentration, reaching a maximum value of 38 nm for M2.8 before reverting to a reduced value for M3.2. This growth of *D* can be attributed to the gradually increasing viscosity of the spraying solution, which influences the spray dynamics. Upon enhancing NH_4_F concentration, the spray solution gets viscous owing to more solute–solvent ion-interaction, which leads to a prolonged residence time of the spray solution on the preheated substrate, allowing for fewer nucleation sites, accompanied by larger crystal growth. R. Ramírez-Amador *et al.* have compared the grown FTO thin films *via* pneumatic spray pyrolysis and ultrasonic spray pyrolysis methods and reported increased crystallinity and enhanced *D* with increasing F/Sn ratio.^37^

The obtained *D* values are further used to calculate the dislocation densities (*δ*) in the prepared FTO thin films employing Williamson and Smallman's method described in eqn (3). Lattice strain (*ε*) is determined using eqn (4). The determined values of *δ* and *ε* are illustrated in Table 2. *δ* can be defined as the length of the dislocation line per unit area, providing an estimate of disorder and crystal structure defects.^38^ It can be observed that the values of *δ* and *ε* generally decrease with increasing dopant salt concentration, reaching their minimum for M2.8, followed by an increase in value for M3.2. This behavior can be attributed to the enhanced crystallinity resulting from increasing NH_4_F concentration, which leads to compact structures and reduced crystal defects. However, further incorporation of fluorine beyond the solubility limit induces additional lattice distortion and micro-strain within the SnO_2_ crystal structure. Moreover, the enhanced tensile strain at M3.2 destabilizes the lattice ordering and promotes defect generation, causing high *δ*. This is consistent with the observed reduction in preferred (200) orientation and the rise in Urbach energy (UE), which will be discussed in the proceeding section, indicating enhanced structural disorder at elevated F levels. On the other hand, the low *δ* and *ε* values of M2.8 are corroborated by its lowest UE value.

**Table 2:** A summary of crystallite size (*D*), dislocation density (*δ*), and lattice strain (*ε*) values of grown FTO thin films as a function of different F/Sn molar ratios

ID	NH_4_F (molar conc.)	*D* (nm)	*δ* (line per nm^2^)	*ε*
M0.4	0.08	18.0	3.1 × 10^−3^	4.65 × 10^−3^
M0.8	0.16	23.0	1.2 × 10^−3^	5.42 × 10^−3^
M1.2	0.24	23.9	1.1 × 10^−3^	3.69 × 10^−3^
M1.6	0.32	24.4	1.7 × 10^−3^	3.40 × 10^−3^
M2.0	0.40	25.0	1.6 × 10^−3^	3.44 × 10^−3^
M2.4	0.48	27.9	1.7 × 10^−3^	3.91 × 10^−3^
M2.8	0.56	38.0	6.9 × 10^−4^	3.12 × 10^−3^
M3.2	0.64	34.1	8.6 × 10^−4^	3.20 × 10^−3^

Raman spectroscopy is employed to gain further insights into the structural properties of the FTO thin films. SnO_2_ tetragonal crystal lattice consists of two tin atoms and four oxygen atoms, which vibrate differently, resulting in various vibrational modes.^36^ The vibrational energies of a molecule give clues about its structure. To observe various Raman active vibrations of SnO_2_, the prepared thin films are scanned in the wavenumber range of 200 to 1200 cm^−1^, as shown in Fig. 1(d). Four peaks were noticed at around 460, 560, 620, and 780 cm^−1^ due to the E_g_, E_u_, A_1g_, and B_2g_ vibrational modes, respectively, validating the tetragonal crystal structure of SnO_2_.^39^ Amongst the vibrations as mentioned above, A_1g_ and B_2g_ are related to the non-degenerate modes and arise from the rotation of the oxygen atom normal to the *c*-axis, while E_g_ is a doubly degenerate vibration originating from along the plane (*c*-axis) movement of oxide ions in the SnO_2_ lattice. Further, A_1g_ corresponds to symmetric, and B_2g_ arises from asymmetric stretching of Sn–O bonds of tetragonal SnO_2_.^40,41^ Geurts *et al.*^42^ observed a similar peak at 782 cm^−1^ in Raman spectra upon oxidation of SnO to SnO_2_. E_u_ infrared active mode is observed at 566 cm^−1^, believed to originate from in-plane oxygen vacancies within the SnO_2_ lattice.^40,41^ A broad peak appearing around 1095 cm^−1^, consistently observed across all samples, can be attributed to the glass substrate used for the FTO deposition.^43,44^ The above findings by XRD and Raman techniques provide strong evidence for the successful development of SnO_2_ tetragonal phase across all prepared FTO samples.

**Fig. 2:**
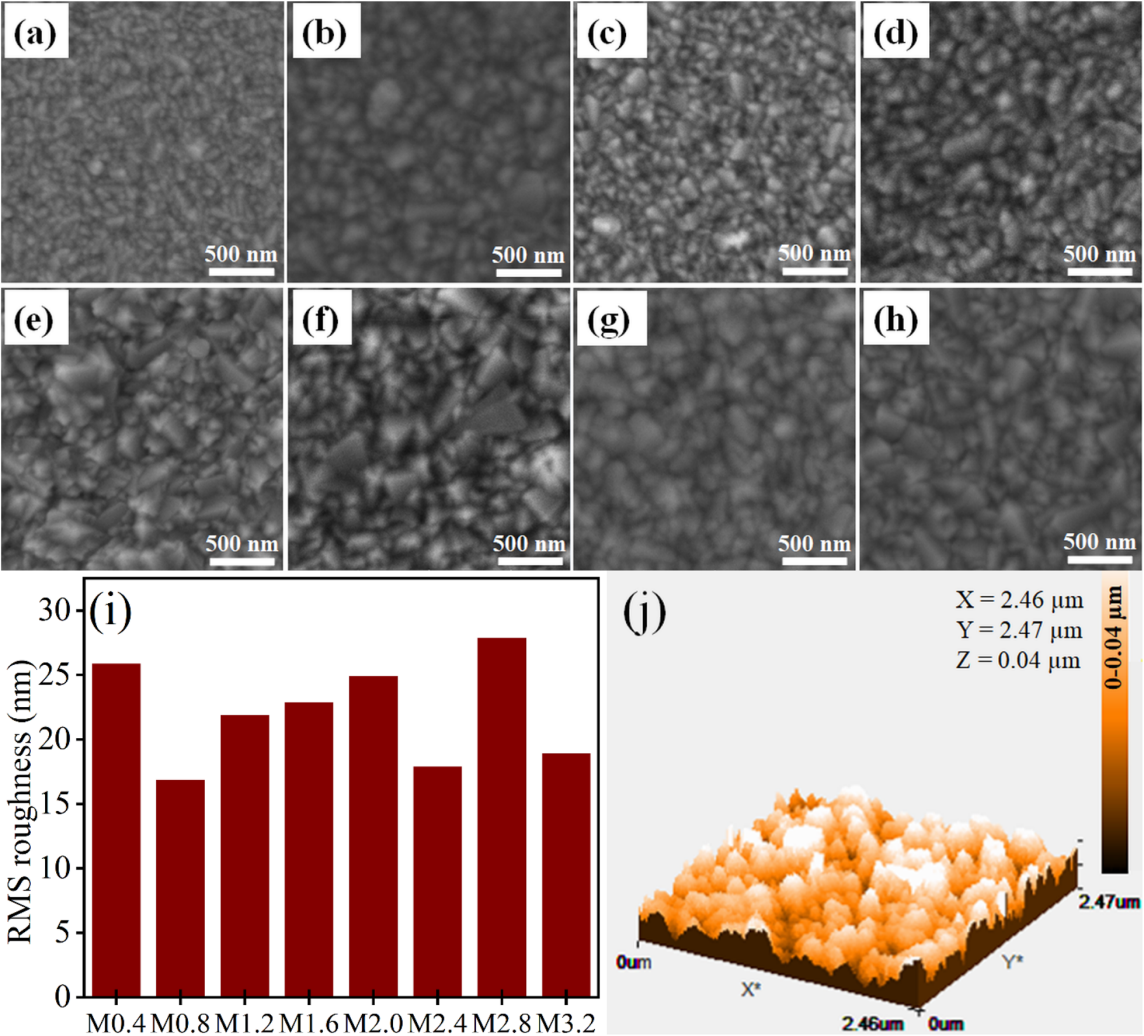
SEM surface images of the prepared FTO thin films upon varying F/Sn molar ratio in the spraying solution; (a) M0.4, (b) M0.8, (c) M1.2, (d) M1.6, (e) M2.0, (f) M2.4, (g) M2.8, (h) M3.2, (i) bar plot of RMS roughness of the FTO thin films as a function of F/Sn molar ratio, and (j) 3D AFM scan of the sample M2.8.

The correlation of surface morphology with varying NH_4_F concentration in the spraying solution in the prepared FTO thin films is analyzed by SEM. Surface micrographs depicted in Fig. 2(a–h) indicate that grown FTO thin films, in general, are highly textured and possess pyramidal and polygon-type grains, as well as smaller bud-like grains. However, a gradual increase in grain size and corresponding improvement in homogeneity of grains can be observed when raising the NH_4_F dopant concentration. This apparent increase in grain size on increasing the F/Sn molar ratio can be attributed to the varying spray dynamics on increasing the viscosity of the spraying solution by adding more NH_4_F. Increasing the viscosity of the spraying solution with the continuous addition of NH_4_F can slow down the evaporation process on the preheated substrate during the deposition of FTO thin films. This slower evaporation extends the stay time of the liquid phase, allowing more time for atom diffusion and reorganization, leading to fewer nucleation sites and promoting the growth of larger grains. Additionally, the thicker thin films are formed (Fig. S3) due to the gradually increasing viscosity of the spraying solution, which further assists in the grain growth. The observed larger pyramidal-type grains can be due to K-{111}^9^ crystal facets, which originate from preferential growth along the (200) direction.^45^ These larger pyramidal grains continuously increased upon increasing dopant concentration, raising the (200) orientation in the prepared FTO thin films, as described in the XRD section. However, smaller grains in between the bigger grains can also be seen in the SEM images that gradually get reduced with an increase in dopant salt concentration. This can be attributed to arise from the (110) orientation.^9,45^ Nevertheless, in contrast to the afore-mentioned studies, a notable trend of TC along (310) is also found in our study, suggesting a similar growth tendency of FTO thin films towards this plane along with (110). These observations are consistent with other studies,^12,25^ where increasing (200) preferred orientation on varying the F/Sn ratio is linked with pyramidal shape grain morphology of FTO thin films. Some other studies, while investigating the effect of varying F/Sn ratio on FTO thin films' morphology, reported increasing grain size upon increasing the F/Sn molar ratio.^24,26^ This micro-structural tuning resulting from improved grain boundaries and crystallinity promotes optoelectronic properties, ultimately boosting overall device performance.^46–48^

The change in thickness of the grown FTO thin films is visible from the SEM cross-sectional images shown in Fig. S3. The variation in FTO thin film thickness as the F/Sn molar ratio changes is plotted in Fig. S4, which clearly shows an increasing trend in thickness as the F/Sn ratio is gradually increased. This can be attributed to the increasing NH_4_F concentration in the spraying solution, which enhances deposition, followed by decomposition, ultimately resulting in a thicker FTO thin film.

The implications of altering the F/Sn ratio in the spraying solution on the surface roughness of the prepared FTO thin films are observed by the AFM analysis. A bar plot of root mean square (RMS) roughness as a function of F/Sn molar ratio is shown in Fig. 2(i), as well as a 3D scan of a representative sample (*i.e.*, optimized sample M2.8, which will be discussed in the following sections) is recorded in the contact mode as shown in Fig. 2(j). AFM scans of other prepared thin film samples are presented in Fig. S5. The corresponding RMS roughness of all thin film samples is found to be increasing with a rising F/Sn ratio; these observations align with previous studies on FTO thin films.^25^ This increased roughness can facilitate light trapping, leading to better efficiency of solar cells.^49,50^

### 3.2. Optoelectronic findings

**Fig. 3:**
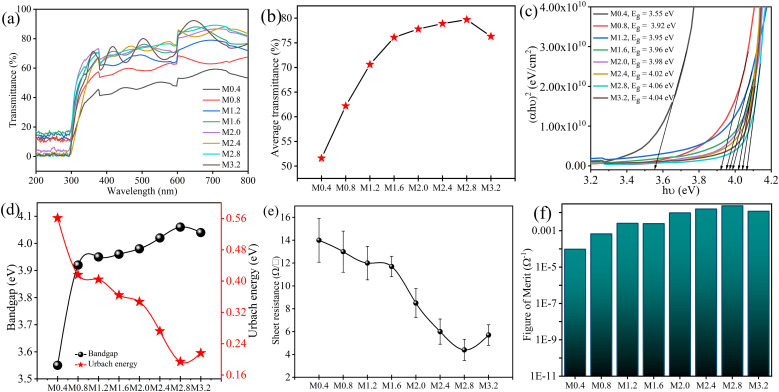
(a) Transmittance profile of the prepared FTO thin films with varying F/Sn ratio, (b) % average transmittance in visible range for the grown thin films, (c) band gaps of prepared FTO thin films determined *via* Tauc plots, (d) a correlation of *E*_g_ and UE of the fabricated FTO thin films, (e) *R*_s_ of FTO thin films along with their corresponding standard deviation represented in the form of error bars (each for 3 replicate samples) in response to the corresponding varying F/Sn ratio, and (f) the *ϕ* values determined by Haackes' eqn (9) for FTO thin films prepared by varying F/Sn ratio.

The transmittance spectra of the prepared FTO thin films are taken in the range of 200–800 nm to look into the effect of varying F/Sn molar ratio in the spraying solution on the optical transmittance behavior of the samples. The sinusoidal pattern in the spectra of grown thin films, as shown in Fig. 3(a), results from interference of light, indicating their uniform thickness. A variation in percentage average transmittance (*T*) in the visible range with an increment of dopant concentration is presented in Fig. 3(b). The *T* values corresponding to samples M0.4, M0.8, M1.2, M1.6, M2.0, M2.4, M2.8, and M3.2 are observed to be 51.6, 62.2, 70.6, 76.1, 77.8, 78.9, 79.7, and 76.3%, respectively. The *T* perpetually increased with increasing NH_4_F concentration up to M2.8, showing a maximum of 83% transmittance in the range of 600–630 nm, and declined on a further increase in concentration. This rise in the *T* with increasing F/Sn molar ratio can be attributed to improved grain boundaries, reduced scattering, and the upward shift in conduction band edges, leading to bandgap (*E*_g_) increase due to a carrier concentration surge, which is consistent with a reported Moss–Burstein phenomenon.^51^

By employing Tauc's eqn (5), the *E*_g_ values of all the prepared thin films are determined, as shown in Fig. 3(c). The *E*_g_ follows a similar trend to *T*; its value ranges from 3.55 eV for M0.4 to a maximum value of 4.06 eV before witnessing a decline for M3.2. This increase in *T* and *E*_g_ of the samples with an increase in doping levels can be further ascribed to the reduced defect states, quantified as Urbach energy (UE) tailing.^52^ UE gives an estimate of the structural disorders in the crystal lattice; it follows an inverse relation with *E*_g_, as vivid in Fig. 3(d), indicating the reduction in crystal defects with increasing crystallinity of prepared FTO thin films owing to increased dopant concentration. These reduced structural defects are also corroborated by decreasing *δ* and *ε* as described in the preceding section. Similar findings of an increase in *E*_g_ with increasing F concentration are reported in the previous study by Karthick *et al.*^12^

The electrical parameters of the FTO thin films in response to changing F/Sn molar ratio in the spraying solution are evaluated using a four-point probe (4pp) and the Hall effect measurement. Sheet resistance (*R*_s_) is determined by using the colinear 4pp method, while Hall effect measurement is performed in the van der Pauw configuration to assess resistivity (*ρ*), carrier concentration (*n*), and mobility (*µ*). The results of these measurements with respect to varying F/Sn ratios are summarized in Table 3. The n-type nature of all FTO thin films, irrespective of the F/Sn ratio, is indicated by the Hall effect measurement. In Fig. 3(e), *R*_s_ values show a decreasing trend from 14 to 4.4 Ω □^−1^ on increasing F/Sn molar ratio in a wide window from 0.4 to 3.2, with the minimum *R*_s_ observed with an F/Sn ratio of 2.8 (*i.e.*, M2.8). This gradual decrease of *R*_s_ can be attributed to the increase of *n* upon raising the F/Sn ratio.

**Table 3:** A summary of electrical parameters, including *ρ*, *n*, and *µ* of the prepared FTO thin films^a^

Sample	*ρ* (ohm cm)	*n* (cm^−3^)	*µ* (cm^2^ V^−1^ s^−1^)
M0.4	9.58 × 10^−3^	5.80 × 10^19^	6.6
M0.8	9.55 × 10^−3^	3.54 × 10^19^	18.0
M1.2	9.41 × 10^−3^	6.35 × 10^19^	25.4
M1.6	9.15 × 10^−3^	7.37 × 10^19^	8.1
M2.0	7.09 × 10^−3^	6.90 × 10^19^	12.8
M2.4	6.05 × 10^−3^	7.18 × 10^19^	25.1
M2.8	1.75 × 10^−3^	1.20 × 10^20^	39.0
M3.2	2.12 × 10^−3^	4.47 × 10^19^	12.5

a *ρ*: resistivity, *n*: charge carriers' concentration, *µ*: mobility.

The value of *n* increases from 5.80 × 10^19^ to 1.20 × 10^20^ cm^−3^ upon increasing the F/Sn ratio from 0.4 to 2.8, accompanied by a declining shift in *ρ*. The increase of *n* and a concomitant drop in *ρ*, as the F/Sn ratio is raised, is linked with enhanced substitutional incorporation of F^−^ in the SnO_2_ lattice.^25^ The *µ* follows a slightly fluctuating pattern as a function of increasing the F/Sn molar ratio, with the highest value being achieved for the M2.8. This *µ*-to-*n* relationship is in contrast to some other reported studies that observed decreasing *µ* with increasing *n*.^12,24^ However, this inter-relationship aligns with some previous studies, presenting the increase of both *µ* and *n*.^53,54^ Ref. 25 in particular, linked a simultaneous surge of both *n* and *µ* with decreased grain boundary scattering. Isshiki *et al.* reported a significantly high *µ* of 77.5 cm^2^ V^−1^ s^−1^ for thin films with carrier concentrations ∼10^20^ cm^−2^.^55^ The improved *µ* despite high *n* can be the outcome of the combined effect of reduced dislocation density,^53^ relaxed lattice strain, and increased texture coefficient along (200),^56^ which has previously been found to have lesser carrier trap states associated with Sn^4+^.^24,57–60^

### 3.3. Figure of merit (*ϕ*)

The figure of merit (*ϕ*) is an important parameter to demonstrate the quality of FTO thin films for photovoltaics and other related applications. Haacke's equation,^32^ described in eqn (9), considers both *T* and *R*_s_ for indexing the quality of TCOs. The *ϕ* values of grown thin films, calculated using *T* (at 550 nm corresponding to the peak sensitivity of the human eye), shown in Fig. 3(f), are found to increase with the rise in the F/Sn ratio in the spraying solution due to an increase in *T* and a concomitant decrease in *R*_s_. The *ϕ* values range from 7.7 × 10^−5^ to 6.1 × 10^−3^ Ω^−1^, with the highest value found to be 2.0 × 10^−2^ Ω^−1^ for M2.8. Table 4 compares the optoelectronic performance and Haack's *ϕ* values of other FTO substrates with the prepared FTO thin films in this study, highlighting the optimized samples' potential for use as a transparent electrode in solar cells and related devices. The observed *ϕ* is comparable and even higher than many other studies reported in the literature.^26,27^ The high transparency of M2.8 allows efficient light transmission, while the low *R*_s_ ensures effective charge carrier transport, making it well-suited for solar cell applications. Based on the various analyses, the optimal sample is M2.8, further tested for thermochemical tolerance testing.

**Table 4:** Comparison of optoelectronic parameters, including *R*_s_, % *T*, and *ϕ* of high-performing spray-pyrolyzed FTO electrodes with the prepared FTO thin films in this study^a^

Year	*R*_s_ (Ω □^−1^)	% *T*	*ϕ* (Ω^−1^)	Reference
2007	7.5	70.0	1.9 × 10^−3^	61
2014	21.0	83.9	7.4 × 10^−3^	62
2017	68.0	78.0	1.2 × 10^−3^	63
2019	9.2	78.6	9.7 × 10^−3^	64
2019	37.4	88.7	8.0 × 10^−3^	64
2025	4.4	79.7	2.0 × 10^−2^	This study

a *R*_s_: sheet resistance, % *T*: transmittance, *ϕ*: figure of merit.

### 3.4. Thermochemical tolerance test

**Fig. 4:**
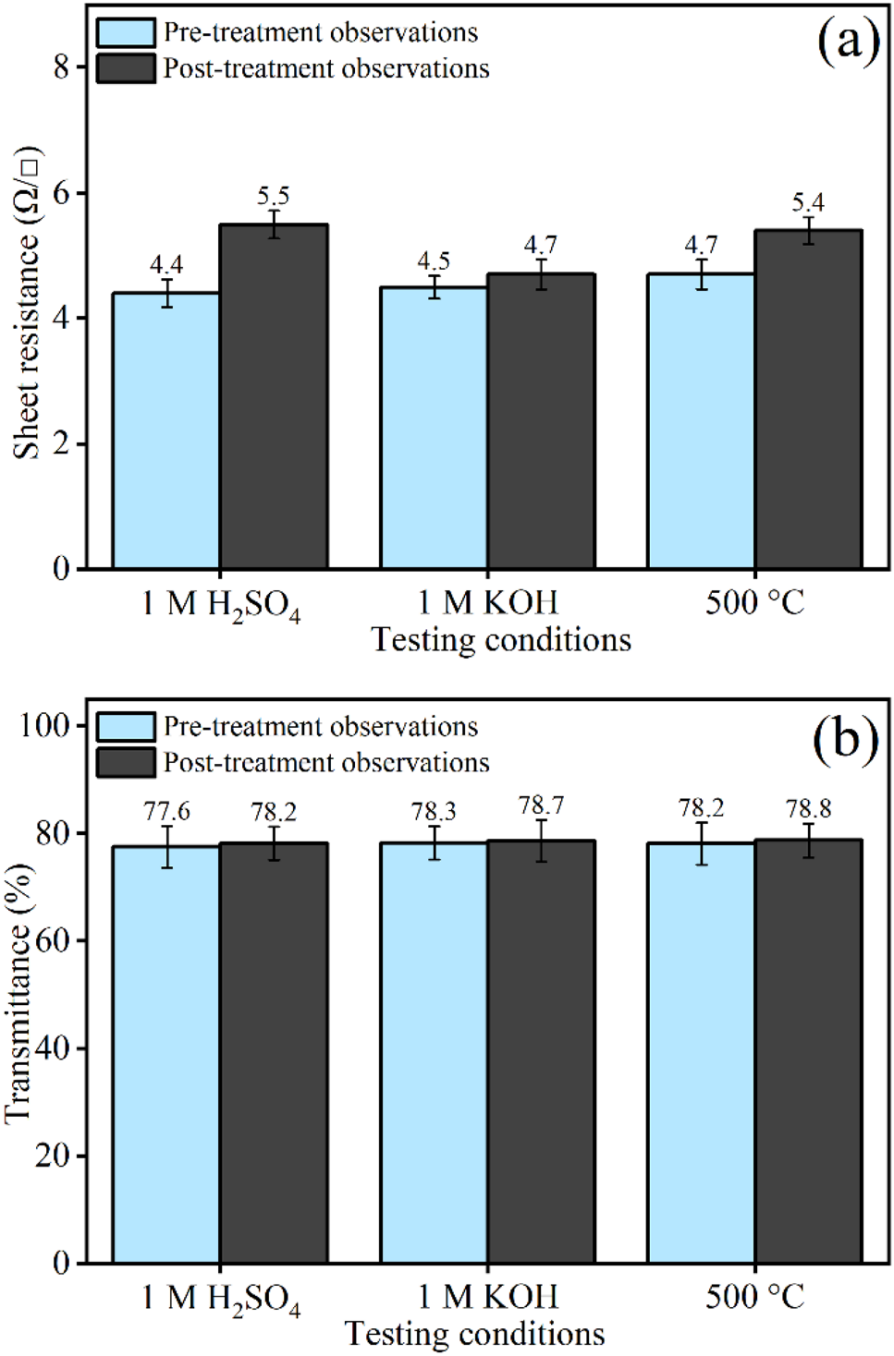
(a) *R*_s_ values and (b) *T* (%) values of best-prepared sample, M2.8, pre- and post-thermochemical treatment.

Thermochemical stability, in addition to the good optoelectronic properties of TCO thin films, is an essential property that needs to be explored when using these as electrode materials in solar cells and other applications. FTO thin films are often considered more thermo-chemically stable compared to other TCOs, such as ITO thin films, due to the doping of a highly electronegative element, fluorine, which creates Sn–F bonds and induces ionic-type bonding within the SnO_2_ lattice.^65^ To assess the thermo-chemical stability of the prepared FTO thin films, the optimized sample, M2.8, is subjected to rigorous thermochemical treatments. Replicate samples of M2.8 are taken for analysis and separately exposed to strong alkaline (1 M KOH) and acidic (1 M H_2_SO_4_) solutions for 72 h, as well as annealed in air at 500 °C for 3 h. After all these three treatments, the thin films are rinsed with distilled water, dried, and analyzed using UV-Vis spectroscopy for optical transmittance and the 4pp method to measure *R*_s_. The bar plots show that there is a negligible change in the *R*_s_ (Fig. 4(a)) and *T* (Fig. 4(b)) values of the samples before and after exposure to all these treatments. Although the change in *R*_s_ values after acidic treatment shows a bit more variation, and this change remains within an acceptable range of standard deviation, therefore, the M2.8 can be relied upon for use under harsh thermochemical conditions. Based on these observations, it can be concluded that M2.8 demonstrates excellent stability under acidic, basic, and thermal conditions, making the sample suitable for applications requiring stability and good optoelectronic characteristics.

### 3.5. Device fabrication

**Fig. 5:**
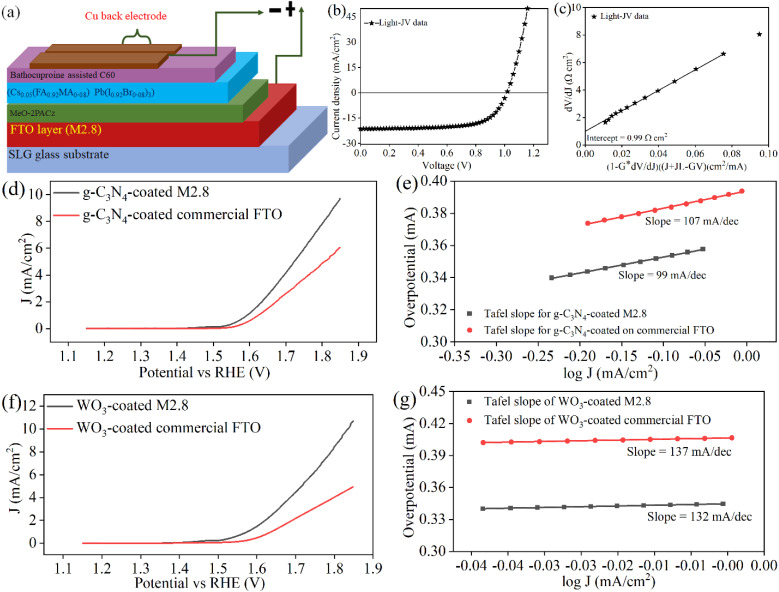
(a) PSC device schematics prepared in an inverted configuration, (b) light-*JV* curve of the representative PSC fabricated on M2.8, (c) a plot of d*V*/d*J versus* (1 − *G* × d*V*/d*J*)(*J* + *JL* − *GV*) is prepared using light-*JV* data provided in (b), (d) comparison of the anodic polarization LSV curves of g-C_3_N_4_-coated-M2.8 and -commercial FTO substrates, (e) corresponding Tafel slope curves, (f) comparison of the anodic polarization LSV curves of WO_3_-coated-M2.8 and -commercial FTO substrates, and (g) corresponding Tafel slope curves.

To demonstrate the utility of the best-prepared FTO thin film in photovoltaic (PV) applications, M2.8 is used to fabricate perovskite solar cells (PSCs) in the inverted configuration, as shown in Fig. 5(a). In the PSC structure, the M2.8 substrate is first coated with self-assembled monolayer MeO-2PACz as a hole transport layer, followed by a perovskite absorber layer of Cs_0.05_(FA_0.92_MA_0.08_)Pb(I_0.92_Br_0.08_)_3_. A bathocuproine-assisted C60 layer is next applied as the electron transport layer, and finally, Cu is deposited as a back electrode. The PV conversion efficiency of the PSC is assessed by acquiring light-*JV* data under standard AM1.5 G illumination. The light-*JV* curve of the representative device is shown in Fig. 5(b), which depicts that the PSC device exhibited an efficiency of 15.0% with an open-circuit voltage of 1.02 V, short-circuit current density of 21.4 mA cm^−2^, and FF of 67%. From the intercept of the plot between d*V*/d*J* on the ordinate and (1 − *G* × d*V*/d*J*)/*J* + *JL* − *GV* on the absicca (Fig. 5(c)), the series resistance of the solar cell is calculated to be 0.99 Ω cm^2^. The series resistance of the fabricated perovskite on M2.8 is significantly low, which is comparable to other high-efficiency solar cells,^66^ underscoring the good quality of the prepared FTO electrodes for photovoltaics.

To further validate the quality of M2.8, the oxygen evolution reaction (OER) performance of g-C_3_N_4_- and WO_3_-coated M2.8 is compared with similarly prepared samples on commercial FTO. OER performance assessment is done in alkaline media (1 M KOH) by employing a three-electrode setup, Ag/AgCl as the reference electrode, graphite rod as the counter electrode, and the prepared substrates as the working electrode. Both the g-C_3_N_4_ and the WO_3_ are known to be effective catalysts for the water oxidation process due to their suitable electronic structure, hydrophilic catalytic surface, low cost, earth-abundant, and enhanced stability.^67,68^ The linear sweep voltammetry (LSV) response depicted in Fig. 5(d and f) illustrates the performance of g-C_3_N_4_- and WO_3_-coated M2.8 and commercial substrates, underscoring the better OER efficiency of the optimized electrode relative to the commercial electrodes. The g-C_3_N_4_-coated M2.8 demonstrates an improved current density (*J*) of 9.7 mA cm^−2^ at a reduced onset potential (*V*_onset_) of 1.517 V (Fig. 5(d)), outperforming the g-C_3_N_4_-coated commercial FTO substrate, which exhibits a *J* of 6.0 mA cm^−2^ and a *V*_onset_ of 1.535 V. Additionally, Tafel slopes, representing a relationship between overpotential and log(*J*) as shown in Fig. 5(e and g), is the indicative of the reaction kinetics of electrodes. It is evident from Fig. 5(d) that g-C_3_N_4_-coated M2.8 has a slightly lower Tafel slope of 99 A dec^−1^ compared with the g-C_3_N_4_-coated commercial FTO substrate, having 107 A dec^−1^.

Likewise, WO_3_-coated M2.8 displays superior efficiency in OER compared with WO_3_-coated commercial FTO thin film, as illustrated in Fig. 5(e). The *J* and *V*_onset_ values of WO_3_-coated M2.8 are 10.7 mA cm^−2^ and 1.501 V, respectively, while for WO_3_-coated commercial FTO, corresponding values are 4.92 mA cm^−2^ and 1.550 V, respectively. Nonetheless, the observed Tafel slope values are 132 A dec^−1^ and 137 A dec^−1^ for WO_3_-coated M2.8 and commercial FTO thin films, showing a negligible difference from each other, as depicted in Fig. 5(g). The observed improved reaction kinetics of the deposited catalytic material exhibited in the case of M2.8 corroborates its on-par performance in comparison to commercial FTO. Further, in both of these test examples, it is clear that g-C_3_N_4_- and WO_3_-coated M2.8 and commercial FTO substrates demonstrate lower *J* values compared with the values obtained when they are deposited on other supports like carbon felt or nickel foam, offering more surface area. However, lower *V*_onset_ and good reaction kinetics are indicative of the good quality of the indigenously grown FTO thin films through facile and cost-effective CSP methods.

## 4. Conclusion

FTO thin films have high demand across different applications, particularly in photovoltaics, light-emitting diodes, smart glass, and gas sensors, due to their good optoelectronic properties and cost-effectiveness compared to other TCOs. In this study, we successfully grew FTO thin films *via* the scalable, cost-effective chemical spray pyrolysis technique by varying the F/Sn molar ratio ranging from 0.4 to 3.2 for the solvent system comprising 90 vol% methanol and 10 vol% water. Optical analysis reveals a steady increase in average % transmittance (*T*), with the M2.8 (*i.e.*, sample grown with F/Sn molar ratio of 2.8) achieving a good *T* of 79%. This improvement is attributed to reduced light scattering and defect states indicated by Urbach energy. Sheet resistance declines, accompanied by an overall increasing charge carrier density, and the highest *µ* is observed for M2.8 to be 39 cm^2^ V^−1^ s^−1^. The figure of merit, a key performance indicator for TCOs, also peaks in M2.8, with a value of 0.02 Ω^−1^. SEM analysis showed better grain growth with a higher F/Sn molar ratio. All thin films showed a preferred orientation along the (200) crystal plane across different F/Sn molar ratios, which is most likely the cause of improved *µ* and reduced grain boundaries. After rigorous testing, the M2.8 sample exhibits excellent thermochemical stability, retaining its optoelectronic properties, including immersion in 1 M H_2_SO_4_ and 1 M KOH for 72 h each, and annealing at 500 °C in air for 3 h. This optimized FTO thin film (M2.8) is used as a substrate for perovskite solar cell fabrication, showing a photovoltaic conversion efficiency of 15% under standard AM1.5 G irradiation. These results emphasize the significant role of optimizing the F/Sn molar ratio in every solvent system.

## Conflicts of interest

There are no conflicts to declare.

## Supplementary Material

RA-016-D5RA08325G-s001

## Data Availability

Data will be made available upon request. Supplementary information (SI) is available. See DOI: https://doi.org/10.1039/d5ra08325g.
