# Supplementary material for: Tuning dopant incorporation in tin oxide thin films using a methanol–water solvent for photovoltaic and water splitting applications
Source: RSC Adv. 2026 Jan 30;16(7):6233–44. doi: 10.1039/d5ra08325g (PMC12856732; doi:10.1039/d5ra08325g)
Supplement: RA-016-D5RA08325G-s001 [file RA-016-D5RA08325G-s001.pdf]

Supplementary Information (SI) for RSC Advances.
This journal is © The Royal Society of Chemistry 2026

Supporting information

Tuning Dopant Incorporation in Tin Oxide Thin Films Using a Methanol–Water Solvent for Photovoltaic and Water Splitting Applications

Musarrat Zahra[a], Shafqat Hussain[b], Khurram Shehzad[c], Ahsan jamal[d], Kashif Yaqub[e], Muhammad Rehan[f], Mohsin Ali Raza Anjum[d], Jaweria Ambreen[a]*, Muhammad Saifullah[d]*

[a]Department of Chemistry, COMSATS University Islamabad, Park Road, 45550, Islamabad, Pakistan.

[b]Physics Division (PD), Pakistan Institute of Nuclear Science and Technology (PINSTECH), Islamabad, Pakistan.

[c]Central Analytical Facility Division (CAFD), Pakistan Institute of Nuclear Science and Technology (PINSTECH), Islamabad, Pakistan.

[d]Chemistry Division (CD), Pakistan Institute of Nuclear Science and Technology (PINSTECH), P.O. Box 45650, Nilore, Islamabad, Pakistan.

[e]LINAC Project, Pakistan Institute of Nuclear Science and Technology (PINSTECH), Nilore 45650, Islamabad, Pakistan

[f] Photovoltaic Research Department, Korea Institute of Energy Research (KIER), Daejeon, South Korea.

Corresponding authors:
Jaweria Ambreen (Jaweria.ambreen@comsats.edu.pk)
Muhammad Saifullah (Saifi.551@gmail.com)

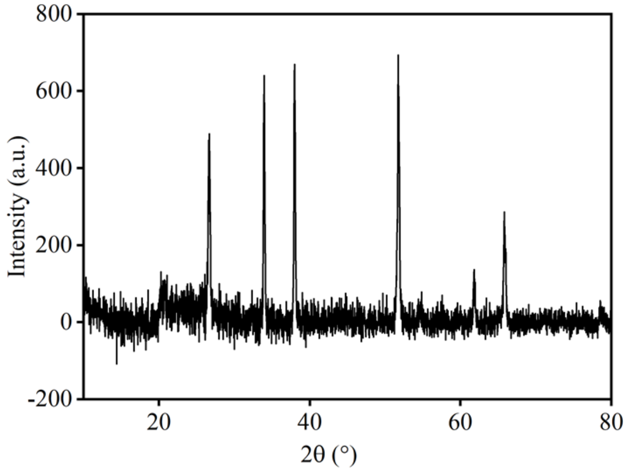

Fig. S1: XRD pattern of WO_3-coated FTO thin film.

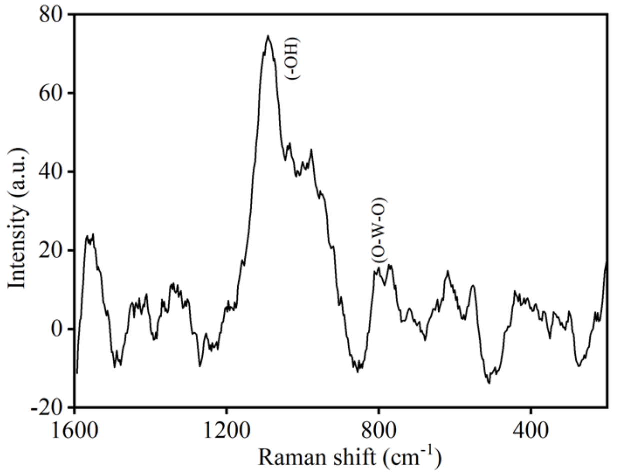

Fig. S2: Raman profile of WO_3-coated FTO thin film.

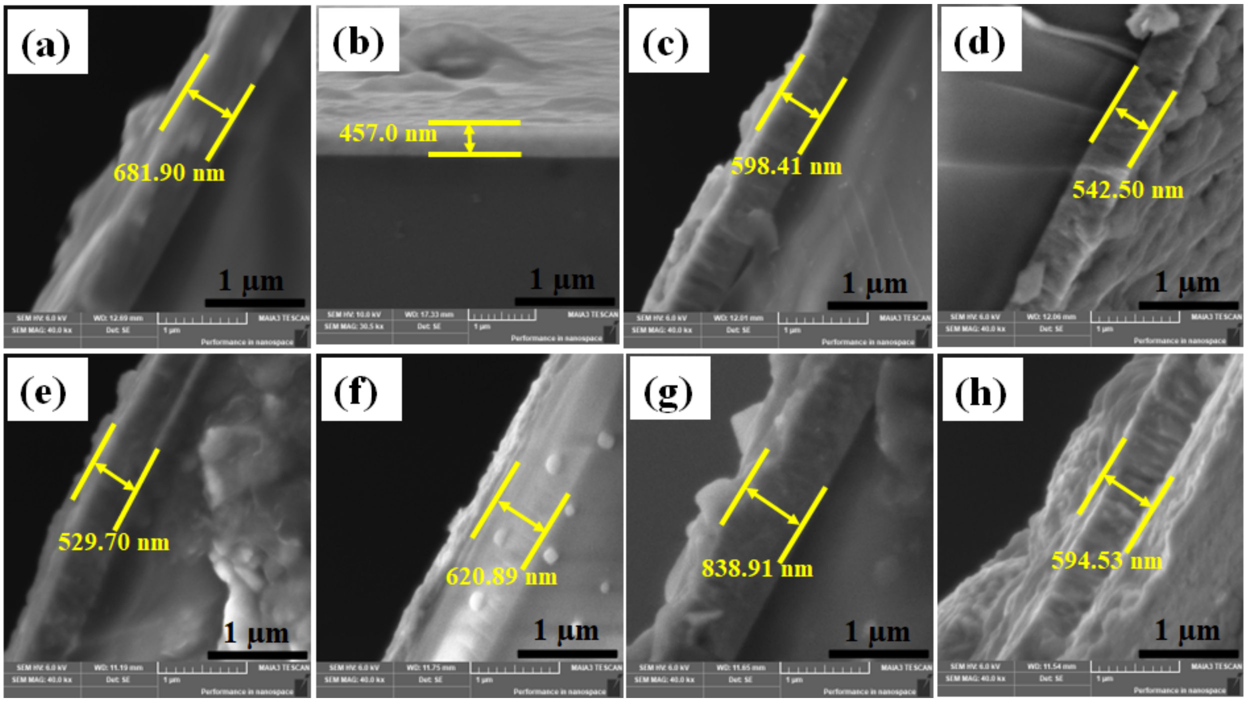

Fig. S3: SEM cross-sectional images of the prepared FTO thin films, (a) M0.4, (b) M0.8, (c) M1.2, (d) M1.6, (e) M2.0, (f) M2.4, (g) M2.8, and (h) M3.2 upon varying F/Sn molar ratio in the spraying solution.

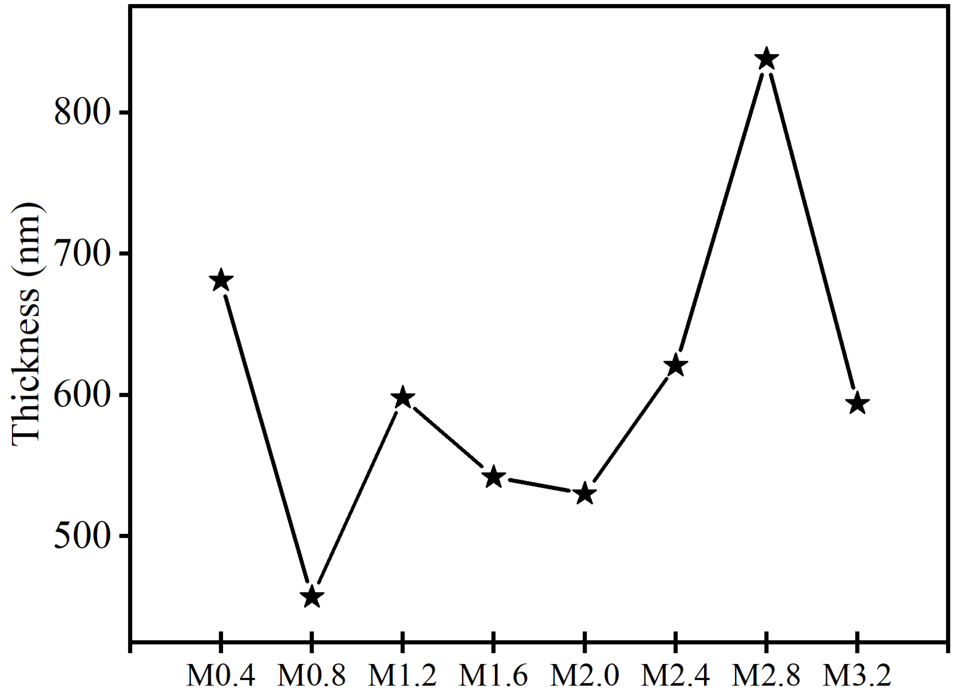

Fig. S4: A correlation of the thickness of FTO thin films with F/Sn molar ratios.

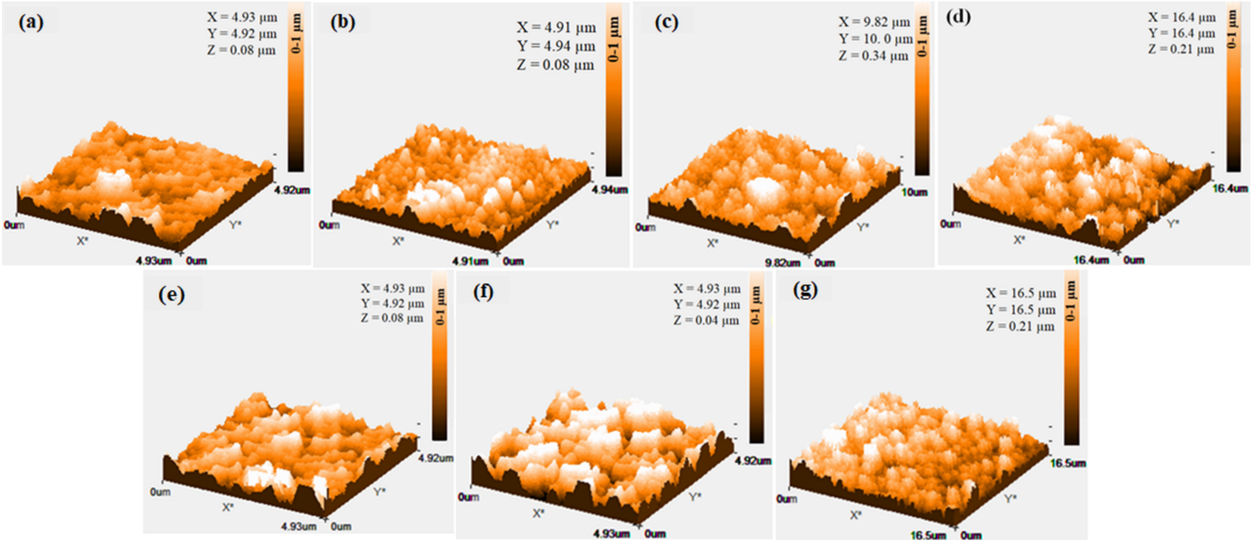

Fig. S5: 3D AFM scans of the prepared FTO thin films upon varying F/Sn molar ratio in the spraying solution; (a) M0.4, (b) M0.8, (c) M1.2, (d) M1.6, (e) M2.0, (f) M2.4, and (g) M3.2.
